# 2-[5-(1,3-Benzodioxol-5-yl)-3-ferrocenyl-4,5-dihydro-1*H*-pyrazol-1-yl]-4-phenyl-1,3-thia­zole

**DOI:** 10.1107/S160053681003638X

**Published:** 2010-09-18

**Authors:** Wei-Yong Liu, Yong-Sheng Xie, Bai-Shan Wang, Bao-Xiang Zhao

**Affiliations:** a Institute of Organic Chemistry, School of Chemistry and Chemical Engineering, Shandong University, Jinan 250100, People’s Republic of China; b Department of Chemical and Environment Engineering, Chongqing Three Gorges University, Chongqing 404100, People’s Republic of China

## Abstract

In the title compound, [Fe(C_5_H_5_)(C_24_H_18_N_3_O_2_S)], the pyrazoline ring adopts a twist conformation. The thia­zole ring forms dihedral angles of 83.7 (2) and 34.4 (2)° with the benzene ring of the benzodioxole ring and the fused phenyl ring, respectively. The mol­ecular conformation is stabilized by an intra­molecular C—H⋯π inter­action. The crystal packing features inter­molecular C—H⋯N, C—H⋯O hydrogen bonds and weak C—H⋯π inter­actions.

## Related literature

For the biological activity of ferrocenyl derivatives, see: Jaouen *et al.* (2004 ▶); Xie *et al.* (2008 ▶, 2010 ▶). For the crystal structures of pyrazoline derivatives, see: Gong *et al.* (2010 ▶). For puckering parameters, see: Cremer & Pople (1975 ▶).
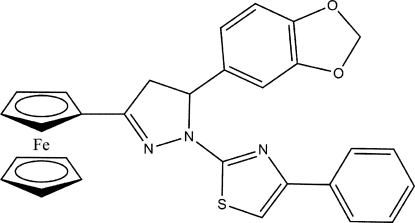


## Experimental

### 

#### Crystal data

[Fe(C_5_H_5_)(C_24_H_18_N_3_O_2_S)]
*M* *_r_* = 533.41
Triclinic, 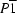

*a* = 10.228 (5) Å
*b* = 11.018 (5) Å
*c* = 12.604 (6) Å
α = 107.776 (8)°
β = 100.416 (8)°
γ = 112.767 (7)°
*V* = 1172.7 (10) Å^3^
*Z* = 2
Mo *K*α radiation
μ = 0.77 mm^−1^
*T* = 293 K
0.15 × 0.10 × 0.10 mm

#### Data collection

Bruker SMART area-detector diffractometer
Absorption correction: multi-scan (*SADABS*; Sheldrick, 1996 ▶) *T*_min_ = 0.894, *T*_max_ = 0.927
6579 measured reflections
4716 independent reflections
3140 reflections with *I* > 2σ(*I*)
*R*_int_ = 0.024

#### Refinement

*R*[*F* ^2^ > 2σ(*F* ^2^)] = 0.050
*wR*(*F* ^2^) = 0.124
*S* = 1.02
4716 reflections
325 parameters
H-atom parameters constrained
Δρ_max_ = 0.30 e Å^−3^
Δρ_min_ = −0.30 e Å^−3^

### 

Data collection: *SMART* (Bruker, 2004 ▶); cell refinement: *SAINT* (Bruker, 2004 ▶); data reduction: *SAINT*; program(s) used to solve structure: *SHELXTL* (Sheldrick, 2008 ▶); program(s) used to refine structure: *SHELXTL*; molecular graphics: *SHELXTL*; software used to prepare material for publication: *SHELXTL*.

## Supplementary Material

Crystal structure: contains datablocks I, global. DOI: 10.1107/S160053681003638X/rz2480sup1.cif

Structure factors: contains datablocks I. DOI: 10.1107/S160053681003638X/rz2480Isup2.hkl

Additional supplementary materials:  crystallographic information; 3D view; checkCIF report

## Figures and Tables

**Table 1:** Hydrogen-bond geometry (Å, °) *Cg*1, *Cg*2, *Cg*3 and *Cg*4 are the centroids of the C13–C18, C25–C29, C1–C6 and C20–C24 rings, respectively.

*D*—H⋯*A*	*D*—H	H⋯*A*	*D*⋯*A*	*D*—H⋯*A*
C15—H15⋯N3^i^	0.93	2.49	3.402 (6)	168
C28—H28⋯O2^ii^	0.98	2.43	3.337 (6)	153
C22—H22⋯*Cg*1^iii^	0.98	2.97	3.709 (5)	133
C26—H26⋯*Cg*1	0.98	2.90	3.844 (5)	163
C5—H5⋯*Cg*2^iv^	0.93	2.91	3.658 (5)	138
C8—H8⋯*Cg*3^v^	0.93	2.98	3.590 (4)	125
C11—H11*A*⋯*Cg*4^vi^	0.97	2.85	3.670 (4)	142

Symmetry codes: (i) 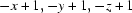; (ii) 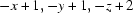; (iii) 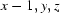; (iv) 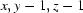; (v) 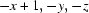; (vi) 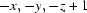.

**Fig. 1.**
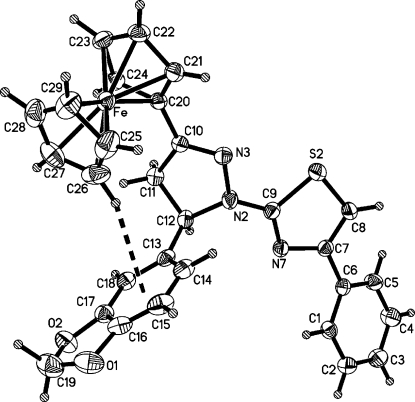
The molecular structure of the title compound showing displacement ellipsoids at the 30% probability level. The intramolecular C—H···π interaction is shown as dashed line.

**Fig. 2.**
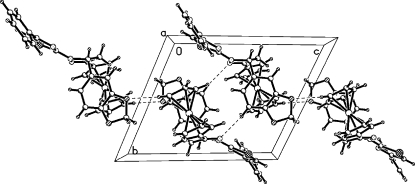
Crystal packing of the title compound viewed along the a axis. Intermolecular hydrogen bonds are shown as dashed lines.

## supplementary crystallographic information

### Comment

Derivatives of pyrazoline possess widespread pharmacological activities. Among them ferrocenyl compounds display interesting antitumor (Jaouen *et al.*, 2004) activities. In our recent study, incorporation of a ferrocene fragment into a heterocyclic ring may enhance their antitumor activities (Xie *et al.*, 2008; Xie *et al.*, 2010, which is rationalized as being due to their different membrane permeation properties and anomalous metabolism. In continuation of previous structural studies of pyrazoline derivatives (Gong *et al.*, 2010), the title compound (I) was synthesized and its crystal structure was determined.

The molecular structure of the title compound is shown in Fig. 1. The conformation of the central pyrazole ring is twist on C11—C12 as indicated by the ring-puckering parameters q2 = 0.204 (4) Å and φ2 = 309.1 (11) ° (Cremer & Pople, 1975), with maximum deviations from the mean plane of the ring of 0.120 (4) and -0.125 (4) Å for atoms C11 and C12, respectively. The thiazole ring forms dihedral angles with the benzene ring of the benzodioxole ring (C13–C18) and the C20–C24 cyclopentadienyl ring of 83.7 (2)° and 47.7 (2)°, respectively, while the dihedral angle between the thiazole and the conjoint phenyl ring (C1–C6) is 34.4 (2)°. The torsion angle C20—*Cg*4—*Cg*2—C26 (*Cg*4 and *Cg*2 are the centroids of the C20–C24 and C25–C29 rings, respectively) of 3.8° indicates an almost eclipsed orientation of two cyclopentadienyl rings. The molecular conformation is stabilized by an intramolecular C—H···π (C26–H26···*Cg*1; Table 1) interaction. In the crystal packing (Fig. 2), zigzag chains are formed through intermolecular C—H···N and C—H···O hydrogen bonds, wherein each molecule is connected to two neighbouring molecules. Furthermore, the structure is stabilized by weak intermolecular C—H···π hydrogen contacts (Table 1).

### Experimental

5-(Benzo[*d*][1,3]dioxol-5-yl)-3-ferrocenyl-4,5-dihydro-1*H*-pyrazole-1-carbothioamide (400 mg, 0.92 mmol), 2-bromo-1-phenylethanone (182 mg, 0.92 mmol) and dichloromethane (8 mL) were added to a round-bottomed flask. The mixture was stirred and heated at reflux under nitrogen for 2 h. The solvent was removed on a rotary evaporator. The residue was purified by column chromatography (silica gel; petroleum ether–EtOAc 3:1  *v*/*v*) to afford title compound. Single crystals suitable for X-ray analysis were obtained by slow evaporation of a solution of the solid in dichloromethane at room temperature for 3 days.

### Refinement

Carbon-bound H-atoms were placed in calculated positions (C—H = 0.93–0.98 Å) and were included in the refinement in the riding model approximation, with *Uiso*(H) set to 1.2 *Ueq*(C).

### Crystal data

**Table:**

[Fe(C_5_H_5_)(C_24_H_18_N_3_O_2_S)]	*Z* = 2
*M**_r_* = 533.41	*F*(000) = 552
Triclinic, *P*1	*D*_x_ = 1.511 Mg m^−^^3^
Hall symbol: -P 1	Mo *K*α radiation, λ = 0.71073 Å
*a* = 10.228 (5) Å	Cell parameters from 1390 reflections
*b* = 11.018 (5) Å	θ = 1.8–26.4°
*c* = 12.604 (6) Å	µ = 0.77 mm^−^^1^
α = 107.776 (8)°	*T* = 293 K
β = 100.416 (8)°	Block, yellow
γ = 112.767 (7)°	0.15 × 0.10 × 0.10 mm
*V* = 1172.7 (10) Å^3^	

### Data collection

**Table:**

Bruker SMART area-detector diffractometer	4716 independent reflections
Radiation source: fine-focus sealed tube	3140 reflections with *I* > 2σ(*I*)
graphite	*R*_int_ = 0.024
phi and ω scans	θ_max_ = 26.4°, θ_min_ = 1.8°
Absorption correction: multi-scan (*SADABS*; Sheldrick, 1996)	*h* = −12→7
*T*_min_ = 0.894, *T*_max_ = 0.927	*k* = −13→13
6579 measured reflections	*l* = −13→15

### Refinement

**Table:**

Refinement on *F*^2^	Primary atom site location: structure-invariant direct methods
Least-squares matrix: full	Secondary atom site location: difference Fourier map
*R*[*F*^2^ > 2σ(*F*^2^)] = 0.050	Hydrogen site location: inferred from neighbouring sites
*wR*(*F*^2^) = 0.124	H-atom parameters constrained
*S* = 1.02	*w* = 1/[σ^2^(*F*_o_^2^) + (0.0498*P*)^2^ + 0.2299*P*] where *P* = (*F*_o_^2^ + 2*F*_c_^2^)/3
4716 reflections	(Δ/σ)_max_ < 0.001
325 parameters	Δρ_max_ = 0.30 e Å^−^^3^
0 restraints	Δρ_min_ = −0.30 e Å^−^^3^

### Special details

**Table:**

Geometry. All e.s.d.'s (except the e.s.d. in the dihedral angle between two l.s. planes) are estimated using the full covariance matrix. The cell e.s.d.'s are taken into account individually in the estimation of e.s.d.'s in distances, angles and torsion angles; correlations between e.s.d.'s in cell parameters are only used when they are defined by crystal symmetry. An approximate (isotropic) treatment of cell e.s.d.'s is used for estimating e.s.d.'s involving l.s. planes.
Refinement. Refinement of *F*^2^ against ALL reflections. The weighted *R*-factor *wR* and goodness of fit *S* are based on *F*^2^, conventional *R*-factors *R* are based on *F*, with *F* set to zero for negative *F*^2^. The threshold expression of *F*^2^ > σ(*F*^2^) is used only for calculating *R*-factors(gt) *etc*. and is not relevant to the choice of reflections for refinement. *R*-factors based on *F*^2^ are statistically about twice as large as those based on *F*, and *R*- factors based on ALL data will be even larger.

### Fractional atomic coordinates and isotropic or equivalent isotropic displacement parameters (Å^2^)

**Table:**

	*x*	*y*	*z*	*U*_iso_*/*U*_eq_	
S2	0.23898 (10)	0.03306 (12)	0.18898 (8)	0.0590 (3)	
Fe	0.13832 (5)	0.39165 (5)	0.68154 (4)	0.04547 (17)	
O1	0.9205 (3)	0.6859 (3)	0.8659 (3)	0.0783 (9)	
O2	0.8642 (3)	0.4884 (3)	0.9074 (2)	0.0666 (7)	
N2	0.3592 (3)	0.1534 (3)	0.4240 (2)	0.0505 (7)	
N3	0.2328 (3)	0.1739 (3)	0.4215 (2)	0.0439 (7)	
N7	0.4875 (3)	0.0646 (3)	0.3137 (2)	0.0418 (6)	
C1	0.7361 (4)	0.0552 (3)	0.2317 (3)	0.0442 (8)	
H1	0.7617	0.1343	0.3008	0.053*	
C2	0.8476 (4)	0.0314 (4)	0.1989 (3)	0.0528 (9)	
H2	0.9483	0.0949	0.2451	0.063*	
C3	0.8108 (4)	−0.0857 (4)	0.0984 (3)	0.0586 (10)	
H3	0.8865	−0.1015	0.0758	0.070*	
C4	0.6632 (4)	−0.1795 (4)	0.0311 (3)	0.0644 (11)	
H4	0.6382	−0.2595	−0.0371	0.077*	
C5	0.5519 (4)	−0.1557 (4)	0.0642 (3)	0.0546 (9)	
H5	0.4514	−0.2211	0.0189	0.065*	
C6	0.5863 (3)	−0.0368 (3)	0.1635 (3)	0.0387 (7)	
C7	0.4688 (3)	−0.0038 (3)	0.1954 (3)	0.0404 (8)	
C8	0.3423 (4)	−0.0305 (4)	0.1179 (3)	0.0526 (9)	
H8	0.3140	−0.0774	0.0361	0.063*	
C9	0.3752 (4)	0.0896 (3)	0.3212 (3)	0.0423 (8)	
C10	0.2057 (3)	0.1734 (3)	0.5167 (3)	0.0368 (7)	
C11	0.3027 (4)	0.1360 (3)	0.5895 (3)	0.0442 (8)	
H11A	0.2493	0.0363	0.5788	0.053*	
H11B	0.3392	0.1992	0.6729	0.053*	
C12	0.4307 (4)	0.1597 (4)	0.5398 (3)	0.0429 (8)	
H12	0.4559	0.0805	0.5287	0.051*	
C13	0.5684 (4)	0.3028 (4)	0.6171 (3)	0.0425 (8)	
C14	0.6077 (4)	0.4218 (4)	0.5902 (3)	0.0534 (10)	
H14	0.5540	0.4115	0.5173	0.064*	
C15	0.7264 (4)	0.5575 (4)	0.6701 (4)	0.0624 (11)	
H15	0.7526	0.6377	0.6521	0.075*	
C16	0.8005 (4)	0.5666 (4)	0.7735 (4)	0.0558 (10)	
C17	0.7652 (4)	0.4490 (4)	0.8002 (3)	0.0476 (8)	
C18	0.6510 (4)	0.3179 (4)	0.7253 (3)	0.0456 (8)	
H18	0.6278	0.2392	0.7452	0.055*	
C19	0.9424 (5)	0.6410 (4)	0.9566 (4)	0.0806 (13)	
H19A	0.9047	0.6804	1.0165	0.097*	
H19B	1.0491	0.6753	0.9936	0.097*	
C20	0.0906 (3)	0.2054 (3)	0.5491 (3)	0.0410 (8)	
C21	0.0226 (4)	0.2786 (4)	0.5058 (3)	0.0522 (9)	
H21	0.0404	0.3125	0.4438	0.063*	
C22	−0.0765 (4)	0.2935 (4)	0.5680 (3)	0.0586 (10)	
H22	−0.1389	0.3405	0.5573	0.070*	
C23	−0.0683 (4)	0.2303 (4)	0.6488 (3)	0.0560 (10)	
H23	−0.1236	0.2266	0.7046	0.067*	
C24	0.0342 (4)	0.1770 (3)	0.6385 (3)	0.0488 (9)	
H24	0.0622	0.1282	0.6847	0.059*	
C25	0.2947 (5)	0.5917 (4)	0.7109 (4)	0.0785 (13)	
H25	0.3093	0.6292	0.6504	0.094*	
C26	0.3654 (4)	0.5192 (4)	0.7469 (5)	0.0796 (14)	
H26	0.4390	0.4969	0.7168	0.096*	
C27	0.3135 (5)	0.4839 (4)	0.8335 (4)	0.0777 (13)	
H27	0.3447	0.4328	0.8756	0.093*	
C28	0.2109 (5)	0.5350 (4)	0.8506 (4)	0.0749 (13)	
H28	0.1571	0.5258	0.9070	0.090*	
C29	0.1981 (5)	0.6019 (4)	0.7753 (4)	0.0716 (12)	
H29	0.1345	0.6486	0.7689	0.086*	

### Atomic displacement parameters (Å^2^)

**Table:**

	*U*^11^	*U*^22^	*U*^33^	*U*^12^	*U*^13^	*U*^23^
S2	0.0488 (5)	0.0842 (7)	0.0444 (6)	0.0376 (5)	0.0140 (4)	0.0201 (5)
Fe	0.0459 (3)	0.0395 (3)	0.0444 (3)	0.0177 (2)	0.0175 (2)	0.0112 (2)
O1	0.0593 (17)	0.0509 (17)	0.104 (2)	0.0132 (14)	0.0179 (17)	0.0294 (18)
O2	0.0615 (16)	0.0570 (17)	0.0603 (18)	0.0191 (13)	0.0054 (14)	0.0186 (14)
N2	0.0580 (18)	0.072 (2)	0.0386 (17)	0.0456 (17)	0.0207 (14)	0.0223 (15)
N3	0.0479 (16)	0.0520 (17)	0.0459 (17)	0.0342 (14)	0.0190 (14)	0.0220 (14)
N7	0.0450 (16)	0.0454 (16)	0.0366 (16)	0.0231 (13)	0.0151 (13)	0.0158 (13)
C1	0.049 (2)	0.0398 (19)	0.0386 (19)	0.0215 (16)	0.0115 (16)	0.0105 (15)
C2	0.046 (2)	0.053 (2)	0.058 (2)	0.0236 (18)	0.0164 (18)	0.0213 (19)
C3	0.058 (2)	0.066 (3)	0.063 (3)	0.041 (2)	0.026 (2)	0.022 (2)
C4	0.061 (2)	0.061 (3)	0.054 (2)	0.034 (2)	0.015 (2)	−0.001 (2)
C5	0.043 (2)	0.051 (2)	0.047 (2)	0.0168 (17)	0.0095 (17)	0.0027 (18)
C6	0.0452 (19)	0.0363 (17)	0.0375 (18)	0.0200 (15)	0.0158 (15)	0.0170 (15)
C7	0.0408 (19)	0.0402 (18)	0.0401 (19)	0.0170 (15)	0.0160 (15)	0.0181 (15)
C8	0.048 (2)	0.071 (3)	0.035 (2)	0.0311 (19)	0.0116 (16)	0.0149 (18)
C9	0.0450 (19)	0.047 (2)	0.042 (2)	0.0249 (16)	0.0191 (16)	0.0202 (16)
C10	0.0369 (17)	0.0317 (17)	0.0383 (19)	0.0159 (14)	0.0104 (14)	0.0119 (14)
C11	0.049 (2)	0.0405 (19)	0.045 (2)	0.0224 (16)	0.0168 (16)	0.0182 (16)
C12	0.054 (2)	0.052 (2)	0.0398 (19)	0.0374 (18)	0.0197 (16)	0.0224 (16)
C13	0.0432 (19)	0.053 (2)	0.051 (2)	0.0326 (17)	0.0251 (17)	0.0278 (18)
C14	0.054 (2)	0.071 (3)	0.068 (3)	0.041 (2)	0.032 (2)	0.048 (2)
C15	0.059 (2)	0.056 (3)	0.096 (3)	0.031 (2)	0.038 (2)	0.051 (3)
C16	0.045 (2)	0.050 (2)	0.081 (3)	0.0234 (18)	0.029 (2)	0.031 (2)
C17	0.046 (2)	0.053 (2)	0.051 (2)	0.0275 (18)	0.0185 (17)	0.0241 (19)
C18	0.049 (2)	0.046 (2)	0.054 (2)	0.0258 (17)	0.0237 (18)	0.0279 (18)
C19	0.074 (3)	0.054 (3)	0.084 (3)	0.024 (2)	0.013 (3)	0.009 (3)
C20	0.0368 (18)	0.0401 (18)	0.0383 (19)	0.0177 (15)	0.0099 (15)	0.0089 (15)
C21	0.049 (2)	0.057 (2)	0.046 (2)	0.0284 (18)	0.0134 (17)	0.0135 (18)
C22	0.046 (2)	0.066 (3)	0.059 (2)	0.0348 (19)	0.0120 (19)	0.012 (2)
C23	0.042 (2)	0.057 (2)	0.059 (2)	0.0179 (18)	0.0243 (18)	0.015 (2)
C24	0.045 (2)	0.0404 (19)	0.054 (2)	0.0158 (16)	0.0194 (17)	0.0161 (17)
C25	0.087 (3)	0.049 (2)	0.079 (3)	0.011 (2)	0.045 (3)	0.018 (2)
C26	0.046 (2)	0.051 (3)	0.097 (4)	0.010 (2)	0.020 (2)	−0.005 (2)
C27	0.076 (3)	0.055 (3)	0.060 (3)	0.019 (2)	−0.007 (2)	0.005 (2)
C28	0.087 (3)	0.057 (3)	0.055 (3)	0.020 (2)	0.028 (2)	0.007 (2)
C29	0.082 (3)	0.042 (2)	0.084 (3)	0.025 (2)	0.040 (3)	0.014 (2)

### Geometric parameters (Å, °)

**Table:**

S2—C8	1.714 (3)	C10—C11	1.488 (4)
S2—C9	1.721 (3)	C11—C12	1.518 (4)
Fe—C28	2.013 (4)	C11—H11A	0.9700
Fe—C27	2.018 (4)	C11—H11B	0.9700
Fe—C20	2.019 (3)	C12—C13	1.505 (5)
Fe—C24	2.020 (3)	C12—H12	0.9800
Fe—C21	2.021 (4)	C13—C14	1.381 (4)
Fe—C29	2.027 (4)	C13—C18	1.392 (4)
Fe—C25	2.028 (4)	C14—C15	1.399 (5)
Fe—C26	2.031 (4)	C14—H14	0.9300
Fe—C22	2.031 (4)	C15—C16	1.337 (5)
Fe—C23	2.031 (4)	C15—H15	0.9300
O1—C16	1.376 (5)	C16—C17	1.367 (5)
O1—C19	1.392 (5)	C17—C18	1.344 (5)
O2—C17	1.359 (4)	C18—H18	0.9300
O2—C19	1.413 (5)	C19—H19A	0.9700
N2—C9	1.345 (4)	C19—H19B	0.9700
N2—N3	1.393 (3)	C20—C21	1.414 (5)
N2—C12	1.478 (4)	C20—C24	1.418 (4)
N3—C10	1.281 (4)	C21—C22	1.417 (5)
N7—C9	1.294 (4)	C21—H21	0.9800
N7—C7	1.388 (4)	C22—C23	1.405 (5)
C1—C2	1.369 (4)	C22—H22	0.9800
C1—C6	1.380 (4)	C23—C24	1.393 (5)
C1—H1	0.9300	C23—H23	0.9800
C2—C3	1.366 (5)	C24—H24	0.9800
C2—H2	0.9300	C25—C26	1.385 (6)
C3—C4	1.365 (5)	C25—C29	1.402 (5)
C3—H3	0.9300	C25—H25	0.9800
C4—C5	1.369 (5)	C26—C27	1.390 (6)
C4—H4	0.9300	C26—H26	0.9800
C5—C6	1.374 (4)	C27—C28	1.389 (6)
C5—H5	0.9300	C27—H27	0.9800
C6—C7	1.471 (4)	C28—C29	1.385 (6)
C7—C8	1.337 (4)	C28—H28	0.9800
C8—H8	0.9300	C29—H29	0.9800
C10—C20	1.443 (4)		
			
C8—S2—C9	88.06 (16)	N2—C12—C11	100.6 (2)
C28—Fe—C27	40.31 (17)	C13—C12—C11	112.1 (3)
C28—Fe—C20	156.29 (17)	N2—C12—H12	110.4
C27—Fe—C20	121.27 (17)	C13—C12—H12	110.4
C28—Fe—C24	120.58 (17)	C11—C12—H12	110.4
C27—Fe—C24	107.07 (17)	C14—C13—C18	119.1 (3)
C20—Fe—C24	41.11 (13)	C14—C13—C12	123.0 (3)
C28—Fe—C21	161.29 (17)	C18—C13—C12	117.6 (3)
C27—Fe—C21	157.07 (18)	C13—C14—C15	121.6 (3)
C20—Fe—C21	40.97 (13)	C13—C14—H14	119.2
C24—Fe—C21	69.01 (15)	C15—C14—H14	119.2
C28—Fe—C29	40.11 (17)	C16—C15—C14	116.9 (3)
C27—Fe—C29	67.72 (19)	C16—C15—H15	121.5
C20—Fe—C29	162.05 (16)	C14—C15—H15	121.5
C24—Fe—C29	155.74 (16)	C15—C16—C17	122.2 (4)
C21—Fe—C29	125.12 (18)	C15—C16—O1	128.3 (4)
C28—Fe—C25	67.40 (18)	C17—C16—O1	109.5 (4)
C27—Fe—C25	67.3 (2)	C18—C17—O2	128.8 (3)
C20—Fe—C25	125.29 (16)	C18—C17—C16	122.0 (4)
C24—Fe—C25	161.49 (17)	O2—C17—C16	109.2 (3)
C21—Fe—C25	108.88 (18)	C17—C18—C13	118.2 (3)
C29—Fe—C25	40.46 (16)	C17—C18—H18	120.9
C28—Fe—C26	67.56 (18)	C13—C18—H18	120.9
C27—Fe—C26	40.16 (18)	O1—C19—O2	108.4 (3)
C20—Fe—C26	108.16 (15)	O1—C19—H19A	110.0
C24—Fe—C26	124.54 (17)	O2—C19—H19A	110.0
C21—Fe—C26	122.27 (18)	O1—C19—H19B	110.0
C29—Fe—C26	67.75 (18)	O2—C19—H19B	110.0
C25—Fe—C26	39.90 (18)	H19A—C19—H19B	108.4
C28—Fe—C22	124.29 (17)	C21—C20—C24	107.9 (3)
C27—Fe—C22	160.44 (19)	C21—C20—C10	127.3 (3)
C20—Fe—C22	68.64 (14)	C24—C20—C10	124.7 (3)
C24—Fe—C22	68.35 (15)	C21—C20—Fe	69.59 (19)
C21—Fe—C22	40.95 (13)	C24—C20—Fe	69.48 (18)
C29—Fe—C22	108.17 (17)	C10—C20—Fe	122.9 (2)
C25—Fe—C22	122.91 (19)	C20—C21—C22	107.5 (3)
C26—Fe—C22	157.9 (2)	C20—C21—Fe	69.4 (2)
C28—Fe—C23	107.49 (17)	C22—C21—Fe	69.9 (2)
C27—Fe—C23	123.92 (19)	C20—C21—H21	126.2
C20—Fe—C23	68.31 (14)	C22—C21—H21	126.2
C24—Fe—C23	40.24 (13)	Fe—C21—H21	126.2
C21—Fe—C23	68.53 (15)	C23—C22—C21	107.9 (3)
C29—Fe—C23	121.52 (16)	C23—C22—Fe	69.8 (2)
C25—Fe—C23	157.59 (18)	C21—C22—Fe	69.2 (2)
C26—Fe—C23	160.5 (2)	C23—C22—H22	126.1
C22—Fe—C23	40.48 (15)	C21—C22—H22	126.1
C16—O1—C19	104.9 (3)	Fe—C22—H22	126.1
C17—O2—C19	105.1 (3)	C24—C23—C22	108.8 (3)
C9—N2—N3	119.2 (3)	C24—C23—Fe	69.4 (2)
C9—N2—C12	125.6 (3)	C22—C23—Fe	69.8 (2)
N3—N2—C12	111.6 (2)	C24—C23—H23	125.6
C10—N3—N2	107.5 (2)	C22—C23—H23	125.6
C9—N7—C7	109.6 (3)	Fe—C23—H23	125.6
C2—C1—C6	120.9 (3)	C23—C24—C20	108.0 (3)
C2—C1—H1	119.6	C23—C24—Fe	70.3 (2)
C6—C1—H1	119.6	C20—C24—Fe	69.41 (18)
C3—C2—C1	119.9 (3)	C23—C24—H24	126.0
C3—C2—H2	120.1	C20—C24—H24	126.0
C1—C2—H2	120.1	Fe—C24—H24	126.0
C4—C3—C2	120.1 (3)	C26—C25—C29	108.5 (4)
C4—C3—H3	120.0	C26—C25—Fe	70.1 (2)
C2—C3—H3	120.0	C29—C25—Fe	69.7 (2)
C3—C4—C5	119.9 (3)	C26—C25—H25	125.8
C3—C4—H4	120.1	C29—C25—H25	125.8
C5—C4—H4	120.1	Fe—C25—H25	125.8
C4—C5—C6	121.0 (3)	C25—C26—C27	107.8 (4)
C4—C5—H5	119.5	C25—C26—Fe	70.0 (2)
C6—C5—H5	119.5	C27—C26—Fe	69.4 (2)
C5—C6—C1	118.2 (3)	C25—C26—H26	126.1
C5—C6—C7	121.7 (3)	C27—C26—H26	126.1
C1—C6—C7	120.1 (3)	Fe—C26—H26	126.1
C8—C7—N7	115.0 (3)	C28—C27—C26	108.0 (4)
C8—C7—C6	125.0 (3)	C28—C27—Fe	69.6 (3)
N7—C7—C6	120.0 (3)	C26—C27—Fe	70.4 (3)
C7—C8—S2	111.2 (3)	C28—C27—H27	126.0
C7—C8—H8	124.4	C26—C27—H27	126.0
S2—C8—H8	124.4	Fe—C27—H27	126.0
N7—C9—N2	124.1 (3)	C29—C28—C27	108.7 (4)
N7—C9—S2	116.1 (2)	C29—C28—Fe	70.5 (2)
N2—C9—S2	119.8 (2)	C27—C28—Fe	70.1 (2)
N3—C10—C20	122.6 (3)	C29—C28—H28	125.7
N3—C10—C11	113.8 (3)	C27—C28—H28	125.7
C20—C10—C11	123.6 (3)	Fe—C28—H28	125.7
C10—C11—C12	102.0 (3)	C28—C29—C25	107.1 (4)
C10—C11—H11A	111.4	C28—C29—Fe	69.4 (2)
C12—C11—H11A	111.4	C25—C29—Fe	69.8 (2)
C10—C11—H11B	111.4	C28—C29—H29	126.5
C12—C11—H11B	111.4	C25—C29—H29	126.5
H11A—C11—H11B	109.2	Fe—C29—H29	126.5
N2—C12—C13	112.5 (3)		

### Hydrogen-bond geometry (Å, °)

**Table:**

Cg1, Cg2, Cg3 and Cg4 are the centroids of the C13–C18, C25–C29, C1–C6 and C20–C24 rings, respectively.

**Table:**

*D*—H···*A*	*D*—H	H···*A*	*D*···*A*	*D*—H···*A*
C15—H15···N3^i^	0.93	2.49	3.402 (6)	168
C28—H28···O2^ii^	0.98	2.43	3.337 (6)	153
C22—H22···Cg1^iii^	0.98	2.97	3.709 (5)	133
C26—H26···Cg1	0.98	2.90	3.844 (5)	163
C5—H5···Cg2^iv^	0.93	2.91	3.658 (5)	138
C8—H8···Cg3^v^	0.93	2.98	3.590 (4)	125
C11—H11A···Cg4^vi^	0.97	2.85	3.670 (4)	142

Symmetry codes: (i) −*x*+1, −*y*+1, −*z*+1; (ii) −*x*+1, −*y*+1, −*z*+2; (iii) *x*−1, *y*, *z*;  (iv) *x*, *y*−1, *z*−1; (v) −*x*+1, −*y*, −*z*; (vi) −*x*, −*y*, −*z*+1.

## References

[bb1] Bruker (2004). *SAINT* and *SMART* Bruker AXS Inc., Madison, Wisconsin, USA.

[bb2] Cremer, D. & Pople, J. A. (1975). *J. Am. Chem. Soc.* **97**, 1354–1358.

[bb3] Gong, Z. L., Zheng, L. W., Zhao, B. X., Yang, D. Z., Lv, H. S., Liu, W. Y. & Lian, S. (2010). *J. Photochem. Photobiol. A*, **209**, 49–55.

[bb4] Jaouen, G., Top, S., Vessireres, A., Leclercq, G., Vaissermann, J. & McGlinchey, M. J. (2004). *Curr. Med. Chem.* **11**, 2505–2517. 10.2174/0929867043364487 15379709

[bb5] Sheldrick, G. M. (1996). *SADABS.* University of Göttingen, Germany.

[bb6] Sheldrick, G. M. (2008). *Acta Cryst.* A**64**, 112–122. 10.1107/S0108767307043930 18156677

[bb7] Xie, Y. S., Pan, X. H., Zhao, B. X., Liu, J. T., Shin, D. S., Zhang, J. H., Zheng, L. W., Zhao, J. & Miao, J. Y. (2008). *J. Organomet. Chem.* **693**, 1367–1374.

[bb8] Xie, Y. S., Zhao, H. L., Su, H., Zhao, B. X., Liu, J. T., Li, J. K., Lv, H. S., Wang, B. S., Shin, D. S. & Miao, J. Y. (2010). *Eur. J. Med. Chem.* **45**, 210–218. 10.1016/j.ejmech.2009.09.046 19879668

